# Real‐world study on fluoropyrimidine‐related toxicity outcomes in cancer patients with select *DPYD* variant alleles that received *DPYD* genotype‐guided dosing

**DOI:** 10.1002/ijc.70005

**Published:** 2025-06-19

**Authors:** Sofía L. J. Peeters, Didier Meulendijks, Zerina Kadric, Sara Ibrovic, Geert‐Jan Creemers, Vanja Milosevic, Matthijs van de Poll, Lieke H. J. Simkens, Birgit A. L. M. Deiman, Hans Gelderblom, Henk‐Jan Guchelaar, Anna M. J. Thijs, Maarten J. Deenen

**Affiliations:** ^1^ Department of Clinical Pharmacy Catharina Hospital Eindhoven The Netherlands; ^2^ Department of Clinical Pharmacy and Toxicology Leiden University Medical Centre Leiden The Netherlands; ^3^ Late Development Oncology AstraZeneca Cambridge UK; ^4^ Department of Medical Oncology Catharina Hospital Eindhoven The Netherlands; ^5^ Department of Clinical Pharmacy Elkerliek Hospital Helmond The Netherlands; ^6^ Department of Clinical Pharmacy Máxima Medical Centre Eindhoven The Netherlands; ^7^ Department of Medical Oncology Máxima Medical Centre Eindhoven The Netherlands; ^8^ Department of Molecular Biology Catharina Hospital Eindhoven The Netherlands; ^9^ Department of Medical Oncology Leiden University Medical Centre Leiden The Netherlands

**Keywords:** 5‐fluorouracil, capecitabine, dihydropyrimidine dehydrogenase, *DPYD*, precision dosing

## Abstract

*DPYD* gene variations are associated with severe fluoropyrimidine toxicity, and an initial 50% dose reduction is widely recommended for heterozygous carriers of relevant *DPYD* variants, including DPYD*2A, DPYD*13, c.2846A>T, and c.1236G>A. However, there is a high variability in DPD activity between *DPYD* variant carriers, and a proportion of patients may tolerate higher fluoropyrimidine doses. The aim of this retrospective study was to compare fluoropyrimidine toxicity outcomes and tolerated dose intensities between different *DPYD* variant carriers that received *DPYD* genotype‐guided dosing. We identified *DPYD* variant carriers that received fluoropyrimidine‐based treatment between January 2015 and February 2021 in three Dutch Hospitals. The initial fluoropyrimidine dose was reduced by 25–50% for all heterozygous *DPYD* variant carriers following the Dutch Pharmacogenetics Working Group guideline. Toxicity outcomes were collected for the first three cycles. From 2112 consecutively *DPYD*‐genotyped patients, 120 patients with *DPYD* variants were included. The frequency of overall severe toxicity was 21% for wild types, 27% for heterozygous *DPYD* variant carriers overall, 19% for c.1236G>A carriers, 38% for c.2846A>T carriers, and 44% for DPYD*2A carriers. Median relative dose intensity for cycles 1–3 was 71% for c.1236G>A carriers, 68% for c.2846A>T carriers, and 52% for DPYD*2A carriers. Despite good fluoropyrimidine tolerance in a large proportion of patients, only 13% of patients underwent dose escalation. Novel studies are highly needed to establish the optimal fluoropyrimidine starting dose for heterozygous carriers of c.1236G>A. After initial dose reduction, dose uptitration based on individual tolerance and therapeutic drug monitoring in all *DPYD* variant heterozygotes is advised to prevent the risk of underdosing.

## Graphical abstract

What's new?

Fluoropyrimidines, a key component of chemotherapy regimens for a variety of solid tumors, can cause severe toxicity. Patients who have specific genetic variants in the *DPYD* gene, which encodes an enzyme that inactivates fluoropyrimidines, have increased susceptibility to this toxicity. A uniform 50% dose reduction is recommended for these patients. However, there is high variability in enzyme activity among different *DPYD* variants. Here, the authors retrospectively assessed fluoropyrimidine tolerance among patients with different *DPYD* variants. The results show that some patients can tolerate doses higher than 50%, particularly those with the c.1236G>A variant. The authors propose that higher fluoropyrimidine doses tailored to individual patients' tolerances could increase treatment efficacy without excessive risk of toxicity.

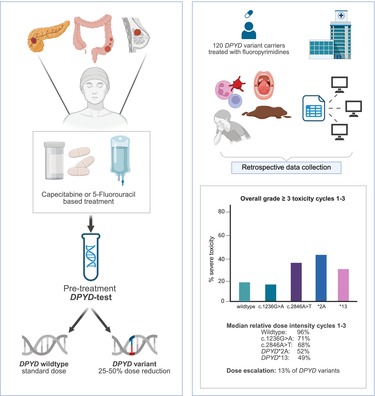

**List of abbreviations**
5‐FU: 5‐fluorouracil
CAPOX: combination treatment with capecitabine and oxaliplatin
CPIC: Clinical Pharmacogenetics Implementation Consortium
CTCAE: Common Terminology Criteria for Adverse Events
DPD: dihydropyrimidine dehydrogenase
DPWG: Dutch Pharmacogenetics Working Group
*DPYD*
: gene encoding DPD
FOLFIRI: combination treatment with 5‐fluorouracil, irinotecan, and folinic acid
FOLFIRINOX: combination treatment with 5‐fluorouracil, oxaliplatin, irinotecan, and folinic acid
FOLFOX: combination treatment with 5‐fluorouracil, oxaliplatin, and folinic acid
RDI: relative dose intensity
SPSS: Statistical Package for the Social Sciences
TDM: therapeutic drug monitoring

## 1 INTRODUCTION

Fluoropyrimidines, such as capecitabine and 5‐fluorouracil (5‐FU), are cornerstone agents in the treatment of various solid tumors, but a substantial challenge lies in the prevention of fluoropyrimidine‐induced severe toxicity that occurs in up to 30% of treated patients. Severe fluoropyrimidine‐related toxicity can result in treatment discontinuation, reduced quality‐of‐life, hospitalization, and in some cases, death.^1^, ^2^, ^3^, ^4^ Fluoropyrimidines are predominantly inactivated through the rate‐limiting enzyme dihydropyrimidine dehydrogenase (DPD). DPD deficiency is therefore known to increase the risk of severe toxicity by two‐ to four‐fold and increase treatment‐related death by 25‐fold due to elevated systemic fluoropyrimidine exposure.^5^, ^6^, ^7^ DPD deficiency is primarily caused by genetic polymorphisms within its encoding gene, *DPYD*, with partial deficiency comprising around 5% of patients.^6^, ^8^, ^9^, ^10^ The most clinically relevant *DPYD* variants include DPYD*2A (c.1905+1G>A, rs3918290), c.2846A>T (rs67376798), DPYD*13 (c.1679T>G, rs55886062), and c.1236G>A (rs56038477, in haplotype B3).^6^, ^11^, ^12^, ^13^, ^14^ Heterozygous carriers of these *DPYD* variants have partial DPD deficiency, and initial fluoropyrimidine dose reductions are crucial to mitigate severe toxicity in these individuals.^5^, ^15^

Upfront *DPYD* screening and *DPYD* genotype‐guided dosing have become widely recommended and used standard care strategies to improve fluoropyrimidine safety.^16^, ^17^, ^18^, ^19^ Current pharmacogenetic guidelines from the Clinical Pharmacogenetics Implementation Consortium (CPIC) and the Dutch Pharmacogenetics Working Group (DPWG) recommend a uniform initial dose reduction of 50% for *DPYD* gene activity scores of 1.0 (DPYD*2A or DPYD*13 heterozygous variant carriers) and 1.5 (c.1236G>A or c.2846A>T heterozygous variant carriers). Patients with a gene‐activity score of 1.5 were earlier recommended treatment with a 25% dose reduction, which was later changed to 50% after the study of Henricks et al. found that an initial 25% dose reduction was insufficient to lower the risk of fluoropyrimidine toxicity in this group.^5^, ^17^, ^19^, ^20^

However, a generalized 50% initial dose reduction for both all heterozygous *DPYD* variant carriers with gene‐activity scores of 1.0 and 1.5 seems counter‐intuitive considering the differences in DPD activity and fluoropyrimidine‐related toxicity between specific *DPYD* variants. The positive predictive value (PPV) of *DPYD* variants to identify patients at‐risk for severe toxicity has been described to typically range between 20 and 85%.^4^, ^12^, ^13^, ^14^ For instance, some *DPYD* variant carriers may tolerate standard doses of fluoropyrimidines without major toxicity.^5^, ^11^, ^21^, ^22^ This is especially true for c.1236G>A variant carriers, a proportion of which have a similar DPD activity and toxicity profile to wild‐type patients.^23^, ^24^, ^25^, ^26^ Differences in effect sizes between the specific *DPYD* variants on DPD activity raise concerns about applying a uniform dose reduction. Without further dose individualization, a uniform dose reduction might lead to underdosing in a proportion of *DPYD* variant carriers with minimally reduced DPD activity.

Consequently, there is a need to study the differences in treatment and toxicity outcomes among clinically relevant *DPYD* variants to enable tailored fluoropyrimidine dosages for further improving treatment outcomes. Therefore, the primary objective of this retrospective study was to compare the frequency of severe fluoropyrimidine‐related toxicity outcomes among patients harboring clinically relevant *DPYD* variants who received *DPYD* genotype‐guided dosing. Secondary objectives included describing the median relative dose intensity (RDI) for *DPYD* variants to provide further insight into tolerated fluoropyrimidine dosages among *DPYD* variant carriers.

## 2 MATERIALS AND METHODS

### 2.1 Study design and patients

We performed a retrospective, multicenter cohort study conducted in three hospitals (Catharina Hospital, Máxima Medical Centre and Elkerliek Hospital) in the Netherlands.

Patients from January 2015 to February 2021 who received at least one cycle of fluoropyrimidine‐based chemotherapy and harbored one of the four clinically relevant *DPYD* variants (DPYD*2A, DPYD*13, c.1236G>A, or c.2846A>T) were enrolled in the study. Patients were genotyped before the start of fluoropyrimidine treatment for the previously mentioned four *DPYD* variants and were treated according to standard fluoropyrimidine‐based anticancer treatment regimens. Additionally, patients were genotyped for variant c.1129‐5923C>G, the causal variant leading to decreased DPD enzyme activity, which is assumed to be in perfect linkage disequilibrium (LD) with variant c.1236G>A. However, rare cases have been reported of patients that only carry c.1236G>A without causal variant c.1129‐5923C>G.^27^ Therefore, patients in the present study were genotyped for both variants. Pre‐treatment *DPYD* testing and *DPYD* genotype guided dosing was hospital policy for every patient starting fluoropyrimidine‐based treatment, with a system‐level infrastructure to support it. In short, *DPYD* test ordering, return of *DPYD* test results, and clinical decision support (CDS) with pre‐ and post‐DPYD test alerts were integrated within the electronic health record (EHR). Test result interpretation and dose reductions were carried out by a medical oncologist and pharmacist and were facilitated by CDS pharmacogenetics consultation notes based on the DPWG guideline. *DPYD* genotyping tests were carried out twice weekly to achieve timely test results. Genotyping was conducted as previously described.^28^ Patients received *DPYD* genotype‐guided dosing based on the *DPYD* guideline of the DPWG using the *DPYD* gene activity score.^17^ Of note, because of changes in the pharmacogenetic guidelines regarding *DPYD* genotype‐guided dosing in the time period of the study cohort,^17^, ^20^ some physicians chose to treat heterozygous c.1236G>A or c.2846A>T variant carriers (gene‐activity score of 1.5) with an initial dose reduction of around 50% (newest guideline recommendation, as per April 2020), while other physicians treated these *DPYD* variant carriers with an initial dose reduction of around 25% (previous guideline recommendation). Heterozygous DPYD*2A or DPYD*13 variant carriers (gene‐activity score of 1.0) received an initial dose reduction of 50%. As is recommended by the DPWG, initial dose reductions could be followed by uptitration in later treatment cycles at the discretion of the treating physician. For comparison, patients who lacked one of the four *DPYD* variants (*DPYD* wild types) and received at least one fluoropyrimidine‐based treatment cycle between January 2018 and October 2019 in the Catharina Hospital were enrolled for a supplementary analysis.

All data on gastrointestinal toxicity (nausea, vomiting, diarrhea, stomatitis, and mucositis) and hematological toxicity (leukocytopenia, neutropenia, and thrombocytopenia) were collected retrospectively from EHRs for the first three treatment cycles and graded according to the National Cancer Institute Common Terminology Criteria for Adverse Events (CTCAE) v4.03. Only toxicities defined as possibly, probably, or definitely related to fluoropyrimidine treatment were collected. Additionally, baseline patient and treatment characteristics were obtained from EHRs. We chose only to include the first three cycles because severe fluoropyrimidine toxicity associated with DPD deficiency typically occurs early during treatment; plus, an analysis of the entire treatment duration was considered inadequate in view of the wide variation among patients in treatment duration and the potential risk of attrition bias.

### 2.2 Endpoints and data analysis

#### 2.2.1 Endpoints

The primary endpoint was overall fluoropyrimidine‐related CTCAE grade ≥3 toxicity during the first three cycles of treatment. Cycle 1–3 toxicity was dichotomized as absent to moderate (CTCAE grade 0–2) versus severe (grade 3–5). Adverse events were merged into categories “overall”, “hematological”, and “gastrointestinal”. Secondary endpoints were fluoropyrimidine RDI, fluoropyrimidine dose reduction or escalation, toxicity‐related hospitalization, treatment delay, treatment discontinuation, and death. Dose modifications were defined as a reduction or escalation of >10% of the administered dose in comparison to the administered dose in the previous treatment cycle. RDI was calculated for the first three treatment cycles by dividing the administered dose (mg/m^2^) in each cycle by the standard dose for *DPYD* wild‐type patients (mg/m^2^) × 100% considering the indication and treatment regimen that was applicable for each patient. Weight change was factored into the calculation of RDI by using the registered weight prior to each treatment cycle for the calculation of BSA (m^2^) and administered dosage (mg/m^2^) of the corresponding cycle.

#### 2.2.2 Data analysis

No formal sample size calculation was performed as the current study was descriptive in nature. Patients were categorized based on *DPYD* variant. Patients treated with chemoradiotherapy and homozygous *DPYD* variant carriers were excluded from the primary analysis and described separately.

Data were described using percentage distributions, means and standard deviations or medians and interquartile ranges, where appropriate. The modified Wald method was used to compute 95% confidence intervals of overall severe toxicity proportions for each *DPYD* variant group. Explorative statistical analyses with a Fisher's exact test were carried out to compare severe toxicity outcomes between *DPYD* variant groups and to compare severe toxicity outcomes between high and low initial fluoropyrimidine dose groups. *p‐*values *p* < .05 were considered statistically significant. Data handling and statistical analyses were performed with SPSS software version 27, and the production of plots was performed with GraphPad Prism software version 9.

## 3 RESULTS

### 3.1 Patient characteristics and genotypes

**FIGURE 1:**
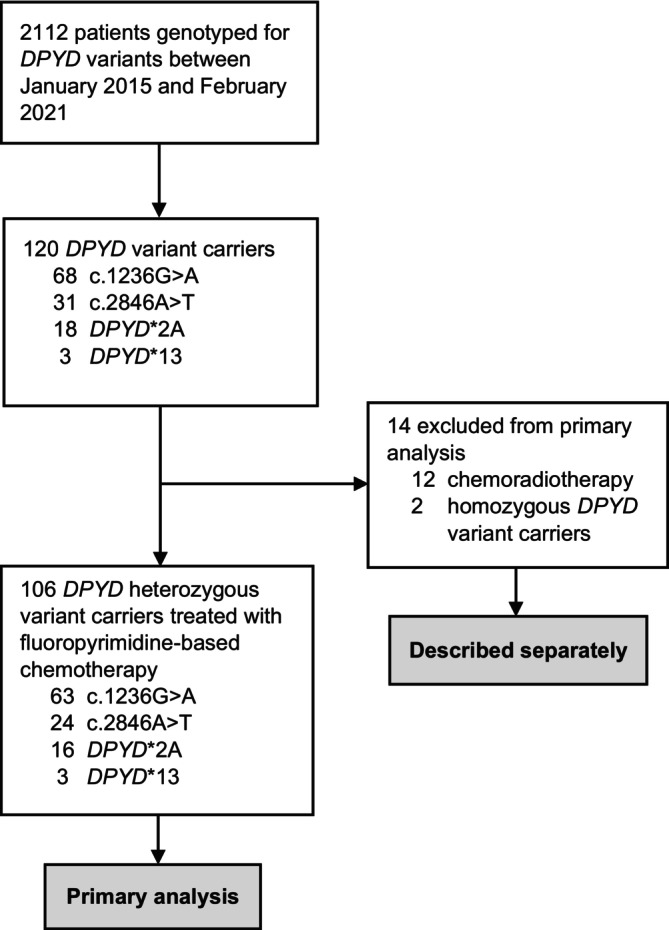
Flow diagram of study population selection.

Between January 2015 and February 2021, 2112 patients were consecutively genotyped for *DPYD*, and a total of 120 patients with *DPYD* variants treated with *DPYD* genotype‐guided dosing of fluoropyrimidines were identified. Among the entire *DPYD* genotyped patient population, the frequency of variant carriers was 3.2% for c.1236G>A, 1.5% for c.2846A>T, 0.85% for DPYD*2A, and 0.14% for DPYD*13. We found that variants c.1236G>A and c.1129‐5923C>G were in perfect LD for all cases harboring a c.1236G>A and c.1129‐5923C>G variant. Out of 120 *DPYD* variant carriers, two patients were homozygous *DPYD* variant carriers, and 12 patients were treated with fluoropyrimidine‐based chemoradiotherapy, leaving 106 heterozygous *DPYD* variant carriers for the primary analysis. We detected no patients with compound heterozygosity. Unintentionally, 12 patients did not or hardly receive an initial fluoropyrimidine dose reduction (initial RDI in first cycle >85%), three of whom received chemoradiotherapy‐based treatment. Figure 1 depicts a selection flow chart of the study selection. The baseline characteristics of the population included in the primary analysis are summarized in Table 1 and baseline characteristics of the *DPYD* wild‐type population are summarized in Table S1.

**TABLE 1:** Baseline patient characteristics of patients included in the primary analysis.

Baseline characteristics	*DPYD* variant carriers (*N* = 106)	c.1236G>A (*N* = 63)	c.2846A>T (*N* = 24)	DPYD*2A (*N* = 16)	DPYD*13 (*N* = 3)
Age (years), median (IQR)	64 (57–72)	64 (57–72)	67 (57–73)	62 (56–68)	69 (63–70)
BSA (m^2^), mean ± STD	1.89 ± 0.20	1.87 ± 0.21	1.90 ± 0.22	1.83 ± 0.24	2.05 ± 0.08
Sex					
Male, *N* (%)	55 (52)	36 (57)	9 (38)	7 (44)	3 (100)
Female, *N* (%)	51 (48)	27 (43)	15 (62)	9 (56)	0 (0)
Ethnicity					
Caucasian, *N* (%)	105 (99)	62 (98)	24 (100)	16 (100)	3 (100)
Non‐Caucasian, *N* (%)	1 (1)	1 (2)	0 (0)	0 (0)	0 (0)
WHO score					
0, *N* (%)	63 (59)	35 (55)	17 (71)	10 (63)	1 (33)
1, *N* (%)	37 (35)	25 (40)	5 (21)	5 (31)	2 (67)
2, *N* (%)	6 (6)	3 (5)	2 (8)	1 (6)	0 (0)
eGFR in mL/min/1,73 m^2^, median (IQR)	85 (71–97)	81 (69–94)	93 (80–97)	93 (72–105)	71 (63–80)
AST in U/L, median (IQR)	25 (20–36)	26 (22–46)	23 (19–31)	25 (18–29)	35 (29–40)
ALT in U/L, median (IQR)	24 (19–40)	24 (20–38)	23 (21–45)	25 (18–39)	23 (22–43)
Primary tumor type					
Colorectal, *N* (%)	61 (57)	34 (54)	14 (59)	11 (69)	2 (67)
Mamma, *N* (%)	18 (17)	11 (17)	6 (25)	1 (6)	0 (0)
Esophagus, *N* (%)	12 (11)	7 (11)	2 (8)	3 (19)	0 (0)
Pancreas, *N* (%)	7 (7)	4 (6)	2 (8)	0 (0)	1 (33)
Stomach, *N* (%)	6 (6)	6 (9)	0 (0)	0 (0)	0 (0)
Other, *N* (%)	2 (2)	1 (2)	0 (0)	1 (6)	0 (0)
Treatment regimen					
Capecitabine monotherapy, *N* (%)	19 (17.9)	14 (22.2)	4 (16.7)	1 (6.3)	0 (0)
Capecitabine + bevacizumab/trastuzumab, *N* (%)	5 (4.7)	4 (6.3)	1 (4.2)	0 (0)	0 (0)
CAPOX, *N* (%)	35 (33.0)	21 (33.3)	4 (16.7)	9 (56.3)	1 (33.3)
CAPOX + bevacizumab/trastuzumab, *N* (%)	23 (21.7)	11 (17.5)	8 (33.3)	3 (18.8)	1 (33.3)
FOLFOX, *N* (%)	6 (5.7)	2 (3.2)	1 (4.2)	3 (18.8)	0 (0)
FOLFOX + bevacizumab, *N* (%)	6 (5.7)	4 (6.3)	2 (8.3)	0 (0)	0 (0)
FOLFIRINOX, *N* (%)	7 (6.6)	4 (6.3)	2 (8.3)	0 (0)	1 (33.3)
Other, *N* (%)^a^	5 (4.7)	3 (4.8)	2 (8.3)	0 (0)	0 (0)
Type of treatment regimen^b^					
Mono chemotherapy, *N* (%)	24 (22.6)	18 (29)	5 (21)	1 (6)	0 (0)
Dual chemotherapy, *N* (%)	75 (70.8)	41 (65)	17 (71)	15 (94)	2 (67)
Triple chemotherapy, *N* (%)	7 (6.6)	4 (6)	2 (8)	0 (0)	1 (33)

*Note*: Data are *N* (%), mean (±STD) or median (IQR).

Abbreviations: ALT, alanine transaminase; AST, aspartate aminotransferase; BSA , body surface area; CAPOX, oral capecitabine combined with oxaliplatin; eGFR, estimated glomerular filtration rate (according to CKD‐EPI); FOLFIRINOX, intravenous 5‐FU combined with oxaliplatin and irinotecan; FOLFOX, intravenous 5‐FU combined with oxaliplatin; IQR, interquartile range; *N*, number of patients; STD, standard deviation.

^a^ Other treatment regimens: capecitabine + carboplatin (*n* = 2), capecitabine + cisplatin + trastuzumab (*n* = 1), capecitabine + temozolomide (*n* = 1), FOLFIRI + bevacizumab (*n* = 1).

^b^ Dual chemotherapy = intravenous 5‐FU or oral capecitabine combined with platinum, or 5‐FU combined with irinotecan; triple chemotherapy = 5‐FU combined with oxaliplatin and irinotecan.

### 3.2 Fluoropyrimidine‐related toxicity in *DPYD* wild‐type patients and heterozygous carriers of variants DPYD*2A, DPYD*13, c.1236G>A, and c.2846A>T

Overall grade ≥3 toxicity was observed in 30 of 144 *DPYD* wild‐type patients (21%; 95% CI: 15–28%) and in 29 of 106 heterozygous *DPYD* variant carriers (27%; 95% CI: 19–37%; *p* = .233). *DPYD* variant carriers showed a comparable frequency of other toxicity‐related endpoints with *DPYD* wild‐type patients (Table S2). Results on toxicity outcomes for each *DPYD* variant are summarized in Table 2. The highest frequency of overall grade ≥3 toxicity was found in DPYD*2A carriers (44%; 95% CI: 23–67%), followed by c.2846A>T carriers (38%; 95% CI: 21–57%) and DPYD*13 carriers (33%; 95% CI: 6–80%). The frequency of overall grade ≥3 toxicity was lowest in c.1236G>A carriers (19%; 95% CI: 11–31%), although differences in overall grade ≥3 toxicity rates between *DPYD* variants were not found to be statistically significant (*p* = .090). Similarly, fluoropyrimidine‐related toxicity resulted in hospitalization for 33% of DPYD*13 carriers, 25% of DPYD*2A carriers, 25% of c.2846A>T carriers, and 10% of c.1236G>A carriers, but differences in hospitalization rates were not statistically significant (*p* = .095). Gastrointestinal grade ≥3 toxicity rates and toxicity‐related treatment discontinuation rates differed significantly between *DPYD* variant groups (*p* = .037 and *p* = .035, respectively) and were lowest in c.1236C>A variant carriers.

**TABLE 2:** Treatment outcomes during the first three fluoropyrimidine‐based treatment cycles for heterozygous *DPYD* variant carriers included in the primary analysis.

Treatment outcomes	*DPYD* variant carriers (*N* = 106)	c.1236G>A (*N* = 63)	c.2846A>T (*N* = 24)	DPYD*2A (*N* = 16)	DPYD*13 (*N* = 3)	*p*‐value^a^
Relative dose intensity first cycle in %						
Median (IQR)	71 (52–75)	72 (53–75)	71 (65–77)	52 (48–54)	48 (NA)	.002
Relative dose intensity cycles 1–3 in %						
Median (IQR)	66 (52–74)	71 (56–75)	68 (62–74)	52 (50–56)	49 (NA)	<.001
Fluoropyrimidine‐related toxicity						
Overall severe grade ≥3 toxicity, *N* (%)^b^	29 (27)	12 (19)	9 (38)	7 (44)	1 (33)	.090
Severe grade ≥3 gastrointestinal toxicity, *N* (%)	25 (24)	9 (14)	9 (38)	6 (38)	1 (33)	.037
Severe grade ≥3 hematological toxicity, *N* (%)	11 (8)	4 (6)	3 (13)	2 (13)	1 (33)	.240
Toxicity‐related hospitalization						
Incidence, *N* (%)	17 (16)	6 (10)	6 (25)	4 (25)	1 (33)	.095
Duration (days), median (IQR)	9 (5–13)	11 (7–14)	8 (5–20)	8 (2–15)	3 (NA)	
Toxicity‐related treatment delay						
Incidence, *N* (%)	17 (16)	9 (14)	6 (25)	2 (13)	0 (0)	.584
Duration (days), median (IQR)	14 (7–27)	21 (7–30)	7 (7–34)	18 (NA)	NA	
Toxicity‐related dose reductions^c^						
Incidence, *N* (%)	12 (11)	6 (10)	4 (17)	2 (13)	0 (0)	.733
Dose escalations^d^						
Incidence, *N* (%)	14 (13)	7 (11)	1 (4)	4 (25)	2 (67)	.027
Toxicity‐related treatment discontinuation						
Incidence, *N* (%)	8 (7)	2 (3)	4 (17)	1 (6)	1 (33)	.035
Toxicity‐related death						
Incidence, *N (%)*	1 (1)	0 (0)	0 (0)	1 (6)	0 (0)	.179

*Note*: Data are *N* (%) or median (IQR).

Abbreviations: IQR, interquartile range; *N*, number of patients; NA, not available.

^a^ *p*‐value comparing heterozygous carriers of c.1236G>A, c.2846A>T, DPYD*2A, and DPYD*13. Fisher's exact test was used for all categorical outcomes, and the Kruskal–Wallis one‐way ANOVA test was used for relative dose intensity outcomes.

^b^ Overall toxicity included gastrointestinal (diarrhea, nausea, vomiting, and mucositis or stomatitis) and/or hematological toxicity (leukocytopenia, neutropenia, and thrombocytopenia).

^c^ Dose reductions were defined as a reduction of >10% of the administered dose in cycle 2 in comparison to the administered dose in cycle 1 and/or a reduction of >10% of the administered dose in cycle 3 in comparison to the administered dose in cycle 2 or 1.

^d^ Dose escalations were defined as an escalation of >10% of the administered dose in cycle 2 in comparison to the administered dose in cycle 1 and/or a reduction of >10% of the administered dose in cycle 3 in comparison to the administered dose in cycle 2 or 1.

One heterozygous DPYD*2A carrier treated with an initial 50% capecitabine dose reduction (CAPOX regimen) died as a result of fluoropyrimidine‐related stomatitis and diarrhea during the second treatment cycle.

Six out of the nine patients that were treated with a full fluoropyrimidine starting dose (initial RDI >85%) developed overall grade ≥3 toxicity (two DPYD*2A, two c.2846A>T, and two c.1236G>A variant carriers). The three other patients (all c.1236G>A variant carriers) that were treated with a full fluoropyrimidine starting dose (initial RDI >85%) did not experience severe fluoropyrimidine‐related toxicity, yet received a dose reduction from the second cycle onward to comply with pharmacogenetic guideline recommendations.

To explore the robustness of the results, a per‐protocol analysis was carried out in which *DPYD* variant carriers treated with a full fluoropyrimidine starting dose (initial RDI >85%) were excluded (Table S3). The per‐protocol analysis yielded comparable results to those of the primary analysis, with slightly lower frequencies of toxicity‐related outcomes: 23 of 97 heterozygous *DPYD* variant carriers (24%) experienced overall grade ≥3 toxicity and c.1236G>A variant carriers demonstrated the lowest frequencies of severe toxicity‐related outcomes (17% overall grade ≥3 toxicity; not statistically significant; Table S3).

### 3.3 Fluoropyrimidine dose escalations in heterozygous carriers of variants DPYD*2A, DPYD*13, c.1236G>A, and c.2846A>T

Fluoropyrimidine dose escalations were attempted during the first three treatment cycles in only 14 heterozygous *DPYD* variant carriers (13%) because of good fluoropyrimidine tolerance (Table 2). In three of these patients, the higher dose resulted in severe toxicity (one DPYD*2A carrier with grade 3 neutropenia after dose escalation from 47% to 63%, one DPYD*2A carrier hospitalized with grade 3 nausea and stomatitis/mucositis after dose escalation from 54% to 80%, and one c.1236G>A carrier hospitalized with grade 3 neutropenia and thrombocytopenia after dose escalation from 76% to 100%). The other 11 patients were able to continue treatment with the escalated dose. 58 *DPYD* variant carriers who completed the first three cycles of fluoropyrimidine treatment without overall grade ≥3 toxicity (55%) did not receive a fluoropyrimidine dose escalation during treatment. Out of this group, 16 patients did not experience any overall fluoropyrimidine‐related toxicity and 42 patients experienced overall grade 1–2 fluoropyrimidine‐related toxicity.

### 3.4 Relative dose intensity trends in heterozygous carriers of variants DPYD*2A, DPYD*13, c.1236G>A, and c.2846A>T

**FIGURE 2:**
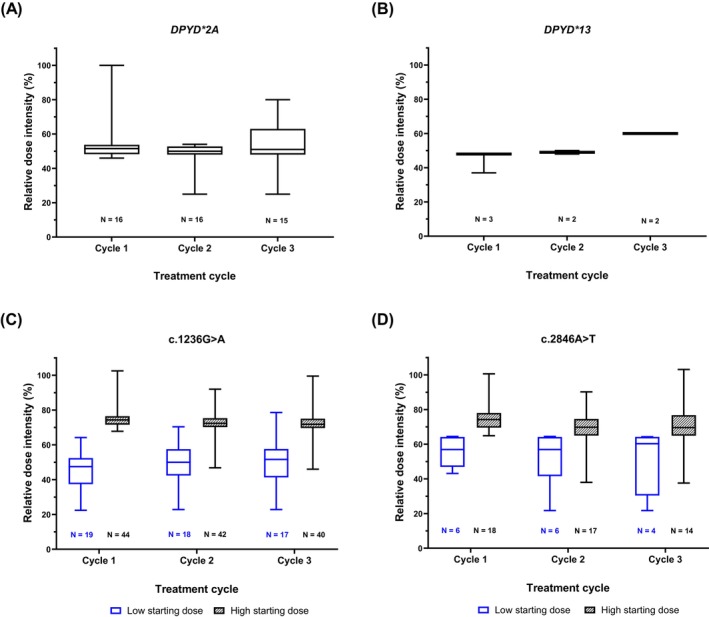
Overview of relative dose intensity during the first three fluoropyrimidine‐based treatment cycles in heterozygous carriers of variants DPYD*2A, DPYD*13, c.1236G>A, and c.2846A>T included in the primary analysis. *N*, number of patients. Heterozygous c.1236G>A and c.2846A>T variant carriers were divided into two groups: low starting dose (relative dose intensity <65% in cycle 1; blue) and high starting dose (relative dose intensity ≥65% in cycle 1; black). (A) Heterozygous carriers of DPYD*2A variant; (B) heterozygous carriers of DPYD*13 variant; (C) heterozygous carriers of c.1236G>A variant; (D) heterozygous carriers of c.2846A>T variant.

**FIGURE 3:**
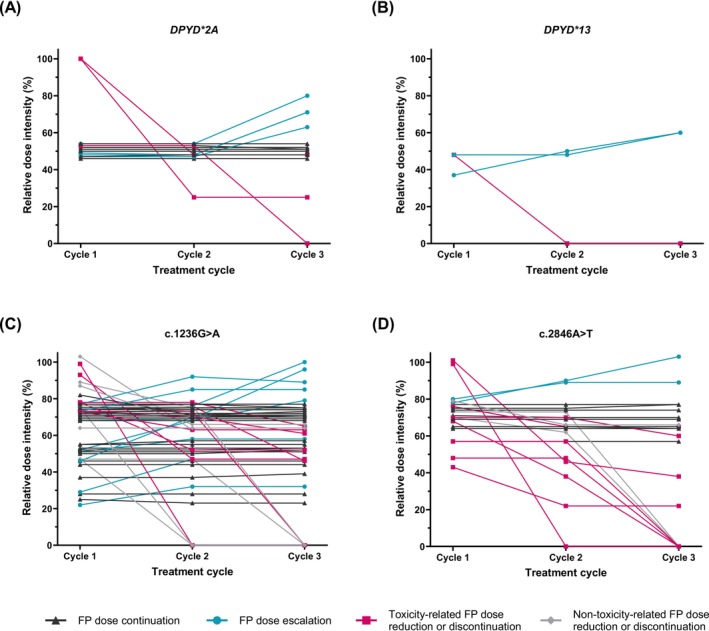
Fluoropyrimidine relative dose intensity trends during treatment cycles 1–3 for individual heterozygous *DPYD* variant carriers included in the primary analysis. Each line represents one patient. FP, fluoropyrimidine. (A) Heterozygous carriers of DPYD*2A variant; (B) heterozygous carriers of DPYD*13 variant; (C) heterozygous carriers of c.1236G>A variant; (D) heterozygous carriers of c.2846A>T variant.

The administered fluoropyrimidine RDI during the first three treatment cycles differed significantly between *DPYD* variants (Table 2). Figure 2 presents an overview of the median RDI during the first three fluoropyrimidine‐based treatment cycles for each specific *DPYD* variant subgroup. Both DPYD*2A variant carriers and DPYD*13 variant carriers remained at a similar median RDI of around 50% during the first three cycles of treatment. Since patients heterozygous for variants c.1236G>A and c.2846A>T received either an initial fluoropyrimidine dose reduction of around 25% or 50%, depending on the treating physician, median RDI was analyzed separately for patients with a high fluoropyrimidine starting dose (cycle 1 RDI ≥65%) and patients with a low fluoropyrimidine starting dose (cycle 1 RDI < 65%). For c.1236G>A carriers with a high starting dose, the median RDI remained similar throughout the first three cycles of treatment (cycle 1: 74% vs. cycle 3: 72%). In addition, c.1236G>A variant carriers with a low starting dose also remained at a similar median RDI during treatment cycles (cycle 1: 47% vs. cycle 3: 52%). For c.2846A>T carriers with a high starting dose, the median RDI decreased slightly from cycle 1 (74%) to cycle 3 (70%) and for c.2846A>T carriers with a low starting dose, the median RDI increased slightly from cycle 1 (57%) to cycle 3 (60%). Moreover, toxicity outcomes of c.1236G>A and c.2846A>T variant carriers were compared between patients with a high fluoropyrimidine starting dose and patients with a low fluoropyrimidine starting dose (Table 3). A higher frequency of patients with a high fluoropyrimidine starting dose experienced overall severe toxicity compared to patients with a low fluoropyrimidine starting dose. For heterozygous c.1236G>A carriers, this was statistically significant for overall grade ≥3 toxicity (0% vs. 27%, respectively; *p* = .01; Table 3). The per‐protocol analysis showed similar results to those of the primary analysis (Table S4). Importantly, a large inter‐patient variability in RDI was observed for c.1236G>A and c.2846A>T carriers, and various of these patients tolerated a fluoropyrimidine RDI of >85% (Figure 2). Figure 3 visualizes individual RDI trends for each *DPYD* variant carrier during the first three fluoropyrimidine‐based treatment cycles.

**TABLE 3:** Toxicity outcomes during the first three fluoropyrimidine‐based treatment cycles for heterozygous c.1236G>A and c.2846A>T variant carriers included in the primary analysis.

Toxicity outcomes	c.1236G>A start RDI < 65% (*N* = 19)	c.1236G>A start RDI ≥ 65% (*N* = 44)	*p*‐value^a^	c.2846A>T start RDI < 65% (*N* = 6)	c.2846A>T high start RDI ≥ 65% (*N* = 18)	*p*‐value^a^
Overall^b^ severe grade ≥3 toxicity, *N* (%)	0 (0)	12 (27)	.01	2 (33)	7 (39)	1.00
Severe grade ≥3 gastrointestinal toxicity, *N* (%)	0 (0)	9 (20)	.05	2 (33)	7 (39)	1.00
Severe grade ≥3 hematological toxicity, *N* (%)	0 (0)	4 (9)	.31	0 (0)	3 (17)	.55
Toxicity‐related hospitalization, *N* (%)	0 (0)	6 (14)	.12	2 (33)	4 (22)	.62

Abbreviations: *N*, number of patients; start RDI, relative dose intensity in cycle 1.

^a^ *p*‐value comparing RDI < 65% group to RDI ≥65%. Fisher's exact test was used.

^b^ Overall toxicity included gastrointestinal (diarrhea, nausea, vomiting, and mucositis or stomatitis) and/or hematological toxicity (leukocytopenia, neutropenia, and thrombocytopenia).

### 3.5 Fluoropyrimidine‐related toxicity outcomes in heterozygous *DPYD* variant carriers treated with chemoradiotherapy

Twelve patients (four heterozygous c.1236G>A variant carriers, six heterozygous c.2846A>T variant carriers, and two heterozygous DPYD*2A variant carriers) received fluoropyrimidine‐based chemoradiotherapy. Median RDI in cycle 1 was 73% (IQR 54–91%). Nine of 12 patients received an initial dose reduction, and one of these patients (DPYD*2A variant carrier) experienced grade ≥3 fluoropyrimidine‐related hematological toxicity during chemoradiotherapy treatment. Three of 12 patients were treated with a full dose of fluoropyrimidines. One of them (DPYD*2A variant carrier) died as a result of fluoropyrimidine‐related stomatitis and diarrhea, and the other two patients (two c.2846A>T variant carriers) did not experience severe fluoropyrimidine‐related toxicity during chemoradiotherapy treatment.

### 3.6 Fluoropyrimidine‐related toxicity outcomes in homozygous *DPYD* variant carriers

Lastly, we identified two homozygous *DPYD* variant carriers who were treated with an initial fluoropyrimidine dose reduction of 50%. One was a homozygous carrier of variant c.1236G>A and did not experience any severe fluoropyrimidine‐related toxicity during the first three cycles of capecitabine and temozolomide combination treatment. The other patient was a homozygous carrier of variant c.2846A>T and died during the first cycle of CAPOX treatment as a result of severe capecitabine‐related stomatitis and neutropenic enterocolitis.

## 4 DISCUSSION

In this study, we provided real‐world data on toxicity outcomes of patients carrying a clinically relevant *DPYD* variant who received *DPYD* genotype‐guided dosing. Our study suggests that fluoropyrimidine tolerance differs between different *DPYD* variant carriers and illustrates the necessity of individual fluoropyrimidine dosing. The prevalence of the four clinically relevant *DPYD* variants together in our study population was 5.7%, which aligns with other European studies^5^, ^29^, ^30^ and highlights the relevance of these variants in the Dutch population.

### 4.1 Toxicity

In our study population, the incidence of overall severe fluoropyrimidine‐related toxicity, defined as CTCAE grade ≥3 gastrointestinal and/or hematological toxicity, was 27% for *DPYD* variant heterozygotes that were treated with *DPYD* genotype‐guided dosing, which is in line with the incidences of 12–39% reported in other real‐world studies of *DPYD* genotype‐guided fluoropyrimidine dosing.^5^, ^26^, ^31^, ^32^, ^33^ It also aligns with the incidence of severe toxicity we found in our group of *DPYD* wild‐type patients (21%) and the incidence described previously for *DPYD* wild‐type patients of 20–30%.^1^, ^5^, ^29^, ^30^, ^34^ We did not calculate relative risks for severe toxicity of the different *DPYD* variants, since these have already been extensively described previously.^5^ Furthermore, 7 out of 12 (58%) *DPYD* variant heterozygotes whose fluoropyrimidine treatment was not guided by *DPYD* genotype developed severe toxicity and one of these patients died. Together, this confirms that fluoropyrimidine‐related toxicity can be extremely severe and that *DPYD* genotype‐guided dosing is vital to reduce severe toxicity risk to baseline level in *DPYD* variant carriers.^15^, ^26^, ^29^, ^30^, ^31^, ^33^

We found a clear trend in fluoropyrimidine tolerance differences between *DPYD* variant genotypes, with c.1236G>A variant carriers seeming to be less prone to experience severe toxicity than DPYD*2A and c.2846A>T variant carriers. Toxicity rates we observed for DPYD*13 variant carriers should be interpreted with caution as only three DPYD*13 variant carriers were included in the present study. Differences in toxicity rates have been reported by multiple previous studies as well^5^, ^22^, ^26^, ^29^, ^30^, ^31^ and can be explained by the differential effect of each variant on DPD activity. c.1236G>A and c.2846A>T have been described to have a more modest effect on DPD enzyme activity (25% and 35% reduced DPD activity, respectively) than variants DPYD*2A and DPYD*13 (45% and 60% reduced DPD activity, respectively).^5^ Heterozygous carriers of variant c.1236G>A especially may therefore be at a smaller risk for developing severe fluoropyrimidine‐related toxicity. Together with our findings, this implies that individual dose‐guiding based on specific *DPYD* genotype is warranted.

### 4.2 Tolerated dose intensity and uptitration

The RDIs that we reported in detail for the first three cycles of treatment reflect the physicians' compliance with the initial fluoropyrimidine dose recommendations by pharmacogenetic *DPYD* dosing guidelines and provide some insights into the tolerated fluoropyrimidine doses of each *DPYD* variant.

Heterozygous carriers of DPYD*2A or DPYD*13 continued on average with a RDI of ~50% during the first three treatment cycles, which is in line with the tolerated dose intensity described previously for DPYD*2A of 48–64%.^5^, ^11^, ^15^, ^21^, ^35^ Although more heterozygous c.1236G>A and c.2846A>T variant carriers with a high fluoropyrimidine starting dose experienced severe fluoropyrimidine‐related toxicity than those with a low starting dose, the majority of c.1236G>A and c.2846A>T carriers could tolerate an initial fluoropyrimidine RDI ≥65% without developing overall severe toxicity (73% of c.1236G>A variants and 61% of c.2846A>T variants). Correspondingly, the median RDI by the third treatment cycle remained around 70% for heterozygous variant carriers of c.2846A>T and c.1236G>A with a high starting dose. This finding aligns with previous studies,^5^, ^21^, ^36^ where the average tolerated RDI was found to be 64% of the standard dose in c.2846A>T variant carriers^5^ and 74–78% in c.1236G>A variant carriers.^5^, ^21^ We were, however, unable to fully validate these tolerated dose intensities in our study population since not all patients received dose escalations in the case of good treatment tolerance.

Interestingly, a substantial number of patients underwent successful fluoropyrimidine dose escalation, even a few DPYD*2A and DPYD*13 heterozygotes. Successful uptitration in *DPYD* variants has been described in other studies^5^, ^29^, ^31^, ^37^ and demonstrates that individual dose‐guiding after initial dose reductions is feasible. We found that multiple patients could tolerate high RDIs of up to 100%, c.1236G>A variant carriers in particular. On the other hand, some *DPYD* variant carriers required further dose reductions because of fluoropyrimidine intolerance, which aligns with the previous finding of Henricks et al. that an initial 25% dose reduction might be insufficient for some c.1236G>A and c.2846A>T carriers.^5^ This high variability in toxicity outcomes between patients within *DPYD* variant groups also suggests that DPD activity, fluoropyrimidine systemic drug exposure, and fluoropyrimidine‐related toxicity cannot be fully predicted by *DPYD* genotype alone.

### 4.3 Implications for clinical practice

Current pharmacogenetic *DPYD* guidelines from CPIC and the DPWG recommend an initial 50% dose reduction in all patients with a DPD activity score of 1.5 (heterozygous carriers of c1236G>A and c.2846A>T) and patients with a DPD activity score of 1.0 (heterozygous carriers of DPYD*2A and DPYD*13),^17^, ^19^ followed by upward dose titration if treatment is well‐tolerated. Yet, the results of this study suggest that the majority of c.1236G>A heterozygous variant carriers can tolerate fluoropyrimidine relative dose intensities above 50%. An initial 50% dose reduction for heterozygous carriers of c1236G>A creates a potential risk of underdosing in patients with little to no decrease in their DPD activity and good treatment tolerance if they are not uptitrated adequately. In the present study, none of the c.1236G>A carriers with a low fluoropyrimidine starting dose experienced severe toxicity, whereas 21% of *DPYD* wild‐type patients with a standard fluoropyrimidine dose experienced severe toxicity, further implying the possibility of underdosing in a proportion of the c.1236G>A carriers. In our study cohort, we did not record efficacy outcomes. A case–control study suggested that in DPYD*2A heterozygotes, 50% dose reduction strategies resulted in similar outcomes compared to *DPYD* wild types.^38^ A recent survival analysis showed that long‐term efficacy outcomes in pooled heterozygous carriers of *DPYD* variants (c.1236G>A, c.2846A>T and DPYD*2A) were not negatively affected by *DPYD* genotype‐guided dosing.^22^ However, for c.1236G>A variant carriers, a shorter progression‐free survival was found, and all c.1236G>A or c.2846A>T heterozygotes received an initial dose reduction of 25%.^22^ The impact of an initial 50% dose reduction on the long‐term survival outcomes of c.1236G>A or c.2846A>T heterozygotes is currently unknown. This underlines the importance of continuous monitoring and individual uptitration where possible, especially for patients in the curative setting. In our study, we found that a number of patients underwent successful uptitration, but the majority of patients were not uptitrated despite having no to mild treatment toxicity. This has been the case in other real‐world studies of *DPYD*‐guided fluoropyrimidine dosing as well^5^, ^22^, ^26^, ^31^, ^32^ and highlights clinician caution in individual uptitration for *DPYD* variants.

### 4.4 Potential solutions and future research

Given the high heterogeneity in fluoropyrimidine tolerance between and within *DPYD* variant subgroups, more work is needed to guide tailored fluoropyrimidine dosing reductions in heterozygous *DPYD* variant carriers.

Currently, therapeutic drug monitoring (TDM) of 5‐FU seems the most valuable dose‐individualization strategy for adjusting 5‐FU dose after initial dose reductions in *DPYD* variants to ensure therapeutic 5‐FU exposure,^37^, ^39^ as is also recommended by CPIC guidelines.^19^ Defined target range recommendations are available for TDM of intravenously administered 5‐FU, and a large number of studies (reviewed in Beumer et al.^39^) have consistently demonstrated that PK‐guided dosing of 5‐FU is feasible and can improve treatment outcomes. Two studies have described successful PK‐guided 5‐FU dose uptitration in patients with a *DPYD* variant.^37^, ^40^ The added value of upfront *DPYD* genotyping combined with PK‐guided 5‐FU dosing as a tool to personalize 5‐FU treatment in *DPYD* variant carriers is currently under further investigation in a Dutch trial.^41^ Unfortunately, TDM evidence and recommendations for capecitabine are currently lacking.

Further studies should be directed at the examination of potential biomarkers that could improve the prediction of severe fluoropyrimidine toxicity in patients who receive *DPYD*‐guided dosing.^42^, ^43^, ^44^, ^45^, ^46^, ^47^ Direct measurement of DPD activity in peripheral blood mononuclear cells for *DPYD* variant carriers may be a useful tool and should especially be considered in patients that are homozygous or compound heterozygous carriers of relevant *DPYD* variants, as is recommended by DPWG guidelines. A downside of DPD phenotyping is that it is limited by its cost‐ and labor‐intensive aspects, and the combined *DPYD* genotyping plus DPD phenotyping strategy still requires further clinical validation.^24^, ^45^, ^48^ Other risk factors for fluoropyrimidine‐related toxicity, such as female sex, age, treatment regimen, and renal function could also be taken into account for guiding initial and subsequent dosing in *DPYD* variant carriers and should be further studied in future larger cohorts.^49^, ^50^, ^51^

Recently, the Association for Molecular Pathology Clinical Practice Committee's Pharmacogenomics Working Group has recommended an extended *DPYD* testing panel that is more representative of genetic diversity across populations.^52^ Therefore, future real‐world studies should investigate how fluoropyrimidine‐related toxicity outcomes and tolerated relative dose intensities of other relevant DPYD variants, such as c.557A>G, c.868A>G, c.2279C>T, DPYD*7, and DPYD*8, relate to the outcomes found in variants DPYD*2A, DPYD*13, c.1236G>A/c.1129‐5923C>G, and c.2846A>T. Moreover, further studies are needed to investigate the effect of integrated haplotypes on severe fluoropyrimidine‐related toxicity. For instance, *DPYD* variants c.85 T>C and c.496A>G have been reported to often be in LD with each other, as well as with c.1236G>A/c.1129‐5923C>G. These variants together, in haplotype combination, may further reduce DPD activity and therefore alter the risk of severe toxicity.^53^ More recently, a common gain‐of‐function genetic variant *DPYD* rs49294451 was found to be associated with a reduced risk of severe fluoropyrimidine‐related toxicity.^54^ *DPYD* rs49294451 has been reported to be in LD with *DPYD* variants c.85 T>C, c.496G>A, and potentially c.1129‐5923C>G/c.1236G>A, but the clinical impact of the haplotype combination of these variants currently remains unknown.

Lastly, future research on real‐world data should elucidate the impact of an initial 50% dose reduction on long‐term efficacy outcomes in heterozygous carriers of c.1236G>A or c.2846A>T. New studies, preferably prospective, should be directed at assessing the optimal fluoropyrimidine starting dose for heterozygous carriers of c.1236G>A in particular, taking into account both toxicity and efficacy outcomes.

### 4.5 Strengths and limitations

This study reflects the real‐life clinical setting of pretreatment *DPYD* testing and *DPYD* genotype‐guided dosing in an unselected group of cancer patients. A limitation of this study is that toxicity data were collected retrospectively from clinical documentation in EHRs, which might have caused registration bias. Furthermore, although reflective of the real‐world setting, the population was heterogeneous, composed of patients with varying tumor types and treatment regimens, thus individual patient‐ and treatment characteristics may have had an additional impact on the observed toxicity rates. Severe toxicity rates may have been confounded by combination chemotherapy regimens with overlapping toxicities and the impact of variants on toxicity may differ between capecitabine and 5‐FU regimens. Eight *DPYD* variant carriers were treated with an irinotecan‐based treatment, 5 of whom were not tested for UGT1A1 enzyme deficiency. However, it is not expected to have a significant impact of reported toxicity outcomes as only 10–15% of patients is a UGT1A1 poor metabolizer. In addition, we cannot rule out that some *DPYD* variants carriers in the present study may have harbored another relevant, unassessed, *DPYD* variant. Considering the low allele frequency of other *DPYD* variants, the chances of compound heterozygosity with other *DPYD* variants in our study population are minimal. However, there may be a possibility of LD with other unassessed *DPYD* variants, which might have an additional impact on DPD enzyme activity and thus on severe toxicity outcomes.^53^, ^54^ Furthermore, no standardized additional dose reductions for patients with renal impairment were applied for *DPYD* variant carriers. Additional initial dose reductions based on hepatic impairment, WHO performance, age or previous treatment‐related toxicity might have been applied in some patients and influenced observed RDIs. Information on this was, however, not collected in the present study. We believe weight change to be have been of limited impact on reported RDIs, as only 22 of 106 *DPYD* variant carriers experienced weight change during the first three cycles and this had no to minimal impact on BSA change and RDI calculations. Finally, our study was descriptive in nature and a post hoc analysis showed that our study was underpowered to detect the currently observed differences in toxicity outcomes between *DPYD* variants. All limitations must be taken into account, and the findings presented in this study must therefore be interpreted with appropriate caution.

## 5 CONCLUSION

In conclusion, this real‐world study reinforces our knowledge that fluoropyrimidine tolerance differs between heterozygous *DPYD* variant carriers and thus dosing recommendations should be adapted based on the type of *DPYD* genetic variant. We found that a relevant proportion of heterozygous c.1236G>A variant carriers can tolerate higher fluoropyrimidine dose intensities than 50% of the standard dose, while having a relatively acceptable rate of severe toxicity. A 50% starting dose for patients carrying the c.1236G>A variant may lead to underdosing in a significant proportion of patients. Therefore, new studies that assess the optimal fluoropyrimidine starting dose for this variant are highly needed. In order to maintain treatment efficacy, initial dose reductions in all heterozygous *DPYD* variant carriers should be followed by closely monitored uptitration based on individual tolerance and TDM if possible. Future studies should investigate methods for safe fluoropyrimidine dose escalation and improved risk stratification of fluoropyrimidine toxicity.

## AUTHOR CONTRIBUTIONS

**Sofía L. J. Peeters:** Conceptualization; investigation; methodology; visualization; formal analysis; writing – original draft; data curation; writing – review and editing. **Didier Meulendijks:** Conceptualization; methodology; investigation; writing – review and editing. **Zerina Kadric:** Investigation; data curation; formal analysis; writing – review and editing. **Sara Ibrovic:** Data curation; investigation; writing – review and editing. **Geert‐Jan Creemers:** Conceptualization; resources; writing – review and editing. **Vanja Milosevic:** Resources; writing – review and editing. **Matthijs van de Poll:** Resources; writing – review and editing. **Lieke H. J. Simkens:** Resources; writing – review and editing. **Birgit A. L. M. Deiman:** Resources; writing – review and editing. **Hans Gelderblom:** Conceptualization; writing – review and editing. **Henk‐Jan Guchelaar:** Conceptualization; methodology; supervision; writing – review and editing. **Anna M. J. Thijs:** Conceptualization; resources; writing – review and editing. **Maarten J. Deenen:** Conceptualization; methodology; investigation; supervision; writing – original draft; writing – review and editing.

## CONFLICT OF INTEREST STATEMENT

D. Meulendijks was an employee of AstraZeneca Farmaceutica, SA, Spain at the time of finalizing this work and a shareholder of AstraZeneca plc, UK. This research received no specific grant from any funding agency in the public, commercial, or not‐for‐profit sectors. The other authors have no conflicts of interest to declare.

## ETHICS STATEMENT

The Medical research Ethics Committee United (MEC‐U, Nieuwegein, the Netherlands) approved the study protocol and declared the study not to be subject to the Medical Research Involving Human Subjects Act (MEC‐U study registration number W19.201). Local approval was obtained from all participating study sites. Given the retrospective character of the study and the anticipated size of the patient population a waiver was provided for informed consent.

## Supporting information


Table S1.

## Data Availability

The data that support the findings of this study are available from the corresponding author upon reasonable request.
