# Supplementary material for: Real‐world study on fluoropyrimidine‐related toxicity outcomes in cancer patients with select DPYD variant alleles that received DPYD genotype‐guided dosing
Source: Int J Cancer. 2025 Jun 19;157(9):1898–911. doi: 10.1002/ijc.70005 (PMC12407033; doi:10.1002/ijc.70005)
Supplement: Supplementary file 1 — Table S1. [file IJC-157-1898-s001.pdf]

Supporting information

MANUSCRIPT:

Real-World Study on Fluoropyrimidine-Related Toxicity Outcomes in Cancer Patients with select *DPYD* Variant Alleles that Received *DPYD* Genotype-Guided Dosing

Sofía Lucía Jacinta Peeters, PharmD; Didier Meulendijks, PharmD, PhD; Zerina Kadric, PharmD; Sara Ibrovic, PharmD; Geert-Jan Creemers, MD PhD; Vanja Milosevic, PharmD; Matthijs van de Poll, PharmD; Lieke H.J. Simkens, MD PhD; Birgit A.L.M. Deiman, PhD; Hans Gelderblom, MD PhD; Henk-Jan Guchelaar, PharmD PhD; Anna M.J. Thijs, MD PhD; Maarten J. Deenen, PharmD PhD

TABLE OF CONTENTS:

Supplementary results

- **Table S1:** Baseline patient characteristics of *DPYD* wildtype patients and of heterozygous *DPYD* variant carriers included in the primary analysis.

- **Table S2:** Treatment outcomes during the first three fluoropyrimidine-based treatment cycles of *DPYD* wildtype patients and of heterozygous *DPYD* variant carriers included in the primary analysis.

- **Table S3:** Treatment outcomes during the first three fluoropyrimidine-based treatment cycles of heterozygous *DPYD* variant carriers included in the per-protocol analysis.

- **Table S4:** Toxicity outcomes during the first three fluoropyrimidine-based treatment cycles of heterozygous c.1236G>A and c.2846A>T variant carriers included in the per-protocol analysis.

Table S1. Baseline patient characteristics of *DPYD* wildtype patients and of heterozygous *DPYD* variant carriers included in the primary analysis.

**Baseline characteristics**	***DPYD variant carriers (N=106)***	***DPYD* wildtype *(N = 144)***
**Age** (years), *median (IQR)*	64 (57-72)	63 (56-70)
**BSA** (m^2), *mean ± STD*	1.89 ± 0.20	1.88 ± 0.24
**Sex**		
Male, *N (%)*	55 (52)	72 (50)
Female, *N (%)*	51 (48)	72 (50)
**Ethnicity**		
Caucasian, *N (%)*	105 (99)	138 (96)
Non-Caucasian, *N (%)*	1 (1)	6 (4)
**WHO-score**		
0, *N (%)*	63 (59)	53 (37)
1, *N (%)*	37 (35)	41 (28)
2, *N (%)*	6 (6)	6 (4)
Unknown, *N (%)*		44 (31)
**eGFR** in ml/ min/1,73 m^2, *median (IQR)*	85 (71-97)	94 (85-97)
**AST** in U/L, *median (IQR)*	25 (20-36)	23 (18-34)
**ALT** in U/L, *median (IQR)*	24 (19-40)	24 (18-41)
**Primary tumor type**		
Colorectal, *N (%)*	61 (57)	70 (49)
Mamma, *N (%)*	18 (17)	13 (9)
Oesophagus, *N (%)*	12 (11)	14 (10)
Pancreas, *N (%)*	7 (7)	20 (14)
Stomach, *N (%)*	6 (6)	22 (15)
Other, *N (%)*	2 (2)	5 (3)
**Treatment regimen**		
Capecitabine monotherapy, *N (%)*	19 (17.9)	16 (11)
Capecitabine + bevacizumab/trastuzumab, *N (%)*	5 (4.7)	7 (5.5)
CAPOX, *N (%)*	35 (33.0)	48 (33)
CAPOX + bevacizumab/trastuzumab, *N (%)*	23 (21.7)	16 (11)
FOLFOX, *N (%)*	6 (5.7)	15 (10)
FOLFOX + bevacizumab/trastuzumab, *N (%)*	6 (5.7)	5 (3.47)
FOLFIRINOX, *N (%)*	7 (6.6)	13 (9)
Other, *N (%)*[a]	5 (4.7)	24 (17)
**Type of treatment regimen**		
Mono chemotherapy, *N (%)*	24 (22.6)	23 (16)
Dual chemotherapy, *N (%)*	75 (70.8)	87 (60)
Triple chemotherapy, *N (%)*	7 (6.6)	34 (24)

Data are N (%), mean (±STD) or median (IQR). BSA = body surface area; eGFR = estimated glomerular filtration rate (according to CKD-EPI); AST = aspartate aminotransferase; ALT = alanine transaminase; STD = standard deviation; IQR = interquartile range; N = number of patients; CAPOX = oral capecitabine combined with oxaliplatin; FOLFOX = intravenous 5-FU combined with oxaliplatin; FOLFIRINOX = intravenous 5-FU combined with oxaliplatin and irinotecan.

[a] Other treatment regimens *DPYD* variants: capecitabine + carboplatin (n=2), capecitabine + cisplatin + trastuzumab (n=1), capecitabine + temozolomide (n=1), FOLFIRI + bevacizumab (n=1). Other treatment regimens *DPYD* wildtypes: FOLFIRINOX-modified (n=6), FOLFOX-bevacizumab with intraperitoneal irinotecan (n=1), Docetaxel, oxaliplatin, capecitabine (DOC) (n=10), 5-FU, oxaliplatin, docetaxel (FLOT) (n=4), FOLFIRI (+bevacizumab) (n=2), capecitabine + vinorelbine (n=1).

Table S2. Treatment outcomes during the first three fluoropyrimidine-based treatment cycles of *DPYD* wildtype patients and of heterozygous *DPYD* variant carriers included in the primary analysis.

Treatment outcomes	*DPYD* variant carriers (*N = 106*)	*DPYD* wildtype (*N = 144*)	*p*-value[c]
**Relative dose intensity first cycle in %**			
Median (IQR)	71 (52-75)	98 (92-100)	<0.001
**Relative dose intensity cycles 1-3 in %**			
Median (IQR)	66 (52-74)	96 (88-100)	<0.001
**Fluoropyrimidine-related toxicity**			
Overall severe grade ≥ 3 toxicity, *N (%)*[a]	29 (27)	30 (21)	0.233
Severe grade ≥ 3 gastrointestinal toxicity, *N (%)*	25 (24)	20 (14)	0.066
Severe grade ≥ 3 haematological toxicity, *N (%)*	11 (8)	15 (10)	1.000
**Toxicity-related hospitalization**			
Incidence, *N (%)*	17 (16)	25 (17)	0.865
**Toxicity-related treatment delay**			
Incidence, *N (%)*	17 (16)	27 (19)	0.618
**Toxicity-related dose reductions**[b]			
Incidence, *N (%)*	12 (11)	26 (18)	0.158
**Toxicity-related death**			
Incidence, *N (%)*	1 (1)	0 (0)	0.424

Data are N (%) or median (IQR). IQR = interquartile range; N = number of patients; NA = not available.

[a] Overall toxicity included gastrointestinal (diarrhea, nausea, vomiting, and mucositis or stomatitis) and/or hematological toxicity (leukocytopenia, neutropenia, and thrombocytopenia).

[b] Dose reductions were defined as a reduction of >10% of the administered dose in cycle 2 in comparison to the administered dose in cycle 1 and/or a reduction of >10% of the administered dose in cycle 3 in comparison to the administered dose in cycle 2 or cycle 1.

[c] p-value comparing heterozygous carriers of c.1236G>A, c.2846A>T, *DPYD**2A and *DPYD**13. Fisher's Exact test was used for all categorical outcomes and Mann-Whitney U Test was used to compare median relative dose intensities.

Table S3. Treatment outcomes during the first three fluoropyrimidine-based treatment cycles of heterozygous *DPYD* variant carriers included in the per-protocol analysis.

Treatment outcomes	*DPYD* variant carriers *(N = 97)*	*c.1236G>A (N = 58)*	*c.2846A>T (N = 22)*	*DPYD*2A (N = 14)*	*DPYD*13 (N = 3)*	*p*-value[d]
**Relative dose intensity first cycle in %**						
Median (IQR)	69 (51-74)	71 (53-75)	70 (65-75)	51 (49-53)	48 (NA)	<0.001
**Relative dose intensity cycles 1-3 in %**						
Median (IQR)	66 (53-74)	71 (58-75)	68 (64-74)	51 (52-54)	49 (NA)	<0.001
**Fluoropyrimidine-related toxicity**						
Overall severe grade ≥ 3 toxicity, *N (%)*[a]	23 (24)	10 (17)	7 (32)	5 (36)	1 (33)	0.236
Severe grade ≥ 3 gastrointestinal toxicity, *N (%)*	19 (20)	7 (12)	7 (32)	4 (29)	1 (33)	0.087
Severe grade ≥ 3 haematological toxicity, *N (%)*	8 (8)	4 (7)	1 (5)	2 (14)	1 (33)	0.196
**Toxicity-related hospitalization**						
Incidence, *N (%)*	13 (13)	5 (9)	4 (18)	3 (21)	1 (33)	0.185
Duration (days), median (IQR)	8 (5-13)	8 (7-13)	7 (5-10)	13 (8-15)	3 (NA)	
**Toxicity-related treatment delay**						
Incidence, *N (%)*	12 (12)	7 (12)	5 (23)	0 (0)	0 (0)	0.239
Duration (days), median (IQR)	14 (7-22)	21 (14-30)	7 (7-7)	NA	NA	
**Toxicity-related dose reductions[b]**						
Incidence, *N (%)*	8 (8)	5 (9)	3 (14)	0 (0)	0 (0)	0.558
**Dose escalations[c]**						
Incidence, *N (%)*	14 (14)	7 (12)	1 (5)	4 (29)	2 (67)	0.017
**Toxicity-related treatment discontinuation**						
Incidence, *N (%)*	6 (6)	1 (2)	3 (14)	1 (7)	1 (33)	0.029
**Toxicity-related death**						
Incidence, *N (%)*	1 (1)	0 (0)	0 (0)	1 (7)	0 (0)	0.175

Data are N (%) or median (IQR). IQR = interquartile range; N = number of patients; NA = not available.

[a] Overall toxicity included gastrointestinal (diarrhea, nausea, vomiting, and mucositis or stomatitis) and/or hematological toxicity (leukocytopenia, neutropenia, and thrombocytopenia). Frequency of overall grade ≥ 3 toxicity: *DPYD**2A: 36% (95% CI: 13%-65%); c.2846A>T: 32% (95% CI: 14-55%); *DPYD**13: 33% (95% CI: 6%-80%), c.1236G>A: 17% (95% CI: 9%-29%).

[b] Dose reductions were defined as a reduction of >10% of the administered dose in cycle 2 in comparison to the administered dose in cycle 1 and/or a reduction of >10% of the administered dose in cycle 3 in comparison to the administered dose in cycle 2 or cycle 1.

[c] Dose escalations were defined as an escalation of >10 % of the administered dose in cycle 2 in comparison to the administered dose in cycle 1 and/or a reduction of >10% of the administered dose in cycle 3 in comparison to the administered dose in cycle 2 or cycle 1.

[d] p-value comparing heterozygous carriers of c.1236G>A, c.2846A>T, *DPYD**2A and *DPYD**13. Fisher's Exact test was used for all categorical outcomes and Kruskal-Wallis one-way ANOVA test was used for relative dose intensity outcomes.

Table S4: Toxicity outcomes during the first three fluoropyrimidine-based treatment cycles of heterozygous c.1236G>A and c.2846A>T variant carriers included in the per-protocol analysis.

Toxicity outcomes	c.1236G>A start RDI < 65% (N = 19)	c.1236G>A start RDI ≥65% (N = 39)	*p*-value[b]	c.2846A>T start RDI < 65% (N = 6)	c.2846A>T high start RDI ≥ 65% (N = 16)	*p*-value[b]
**Overall[a] severe grade ≥ 3 toxicity, *N (%)***	0 (0)	10 (26)	0.022	2 (33)	5 (31)	1.000
**Severe grade ≥ 3 gastrointestinal toxicity, *N (%)***	0 (0)	7 (18)	0.084	2 (33)	5 (31)	1.000
**Severe grade ≥ 3 haematological toxicity, *N (%)***	0 (0)	4 (11)	0.291	0 (0)	1 (6)	1.000
**Toxicity-related hospitalization, *N (%)***	0 (0)	5 (13)	0.161	2 (33)	2 (12)	0.292

N = number of patients; start RDI = relative dose intensity in cycle 1.

[a]Overall toxicity included gastrointestinal (diarrhea, nausea, vomiting, and mucositis or stomatitis) and/ or hematological toxicity (leukocytopenia, neutropenia, and thrombocytopenia).

[b]p-value comparing RDI < 65% group to RDI ≥ 65%. Fisher's Exact Test was used.
